# The causal effects of genetically predicted sex hormone-binding globulin and sarcopenia: A 2-sample Mendelian randomization study

**DOI:** 10.1097/MD.0000000000044654

**Published:** 2025-09-26

**Authors:** Moyan Liu, Lichao Zhao, Yan Gao, Xiaoping Sun, Jie Xu, Juan Hui, Peng Xu, Qingfeng Yu, Zhaoyan Zhang

**Affiliations:** a First Department of Cadre Ward, The 960th Hospital of the PLA Joint Logistic Support Force, Jinan, China; b Department of Health Care, The 960th Hospital of the PLA Joint Logistic Support Force, Jinan, China; c Department of General Practice, The 960th Hospital of the PLA Joint Logistic Support Force, Jinan, China.

**Keywords:** GWAS, Mendelian randomization, sarcopenia, sex hormone-binding globulin, SHBG

## Abstract

Observational studies suggest a link between sex hormone-binding globulin (SHBG) and sarcopenia, but the causal relationship is unclear. We conducted a 2-sample Mendelian randomization (MR) study using genome-wide association studies summary data to explore the causal associations between SHBG and traits of sarcopenia, including appendicular lean mass, hand grip strength, and walking pace. Our primary method was inverse-variance weighted, supplemented by weighted median, weighted mode, and MR-Egger regression. Horizontal pleiotropy was assessed using MR-Egger and Mendelian randomization-Pleiotropy Residual Sum and Outlier, with a leave-one-out analysis for robustness. Inverse-variance weighted results showed that there was a causal relationship between genetically predicted SHBG and usual walking pace (odds ratio = 1.04057, 95% confidence interval: 1.01542–1.06635, *P* = .00144). The results of our study indicate a causal association between SHBG and sarcopenia. Further research is required to confirm these observations and better understand the underlying mechanisms linking SHBG to sarcopenia.

## 1. Introduction

Sarcopenia is a systemic skeletal muscle disorder marked by a gradual decline in muscle mass and functionality.^[1–3]^ It can be primary, primarily linked to aging, or secondary, occurring alongside various chronic conditions in middle aged individuals, including inflammation, cancer, or organ malfunction.^[2,4]^ Globally, it is estimated that sarcopenia impacts between 10% and 16% of elderly adults.^[1]^ Women and people with chronic medical conditions are more likely to develop sarcopenia.^[5]^ As the world’s population ages, the incidence of sarcopenia is rising rapidly, and by 2050 it is expected that 500 million people will suffer from the disease.^[6,7]^ Sarcopenia is linked to an elevated risk of numerous detrimental health consequences. These include reduced overall and progression-free survival rates, increased postoperative complications and extended hospital stays among patients with various medical conditions.^[8,9]^ Additionally, it is associated with falls and fractures, metabolic disturbances, cognitive decline, and increased mortality in the general population.^[1]^

Sex hormone-binding globulin (SHBG) is a serum protein with a strong affinity for binding to circulating sex hormones. Alterations in SHBG levels can lead to a range of physiological and pathological effects within the human body.^[10,11]^ Studies suggest that the mechanism of the association between SHBG and sarcopenia is related to changes in testosterone concentration in the body.^[1,12]^ Testosterone is an important anabolic hormone that is closely associated with changes in body composition and circulates primarily in the plasma in albumin binding or SHBG bound or free form.^[1]^ Changes in SHBG concentrations do not affect the physiological properties and biological effects of androgens in the normal homeostatic environment of the organism. A specific increase in SHBG concentration results in an increase in serum testosterone concentration, at which point testosterone is mostly circulating as albumin binding or SHBG, with only a small fraction (1–4%) circulating freely in the plasma.^[13,14]^ Testosterone plays an important role in maintaining muscle mass and bone mineral density in middle aged and elderly men, promoting hematopoiesis, inhibiting lipogenesis and increasing lipolysis.^[15,16]^ Studies have shown that low serum testosterone levels are associated with osteoporosis, insulin resistance, as well as sarcopenia and increased fat mass.^[17–19]^ However, the relationship between SHBG and sarcopenia has not been clearly explained to date, and the exact mechanism of the relationship between SHBG and sarcopenia remains unclear.

In this research, we utilized Mendelian randomization (MR) analysis, leveraging genetic variations as instrumental variables (IVs) to deduce causality between an exposure and an outcome. This approach was chosen because genetic variants, specifically single nucleotide polymorphisms (SNPs), are independent of confounding factors and reverse causation.^[20]^ The principles of MR studies are based on Mendelian laws, similar to randomized controlled trials while avoiding high costs.^[21]^ In epidemiological research, the presence of confounding factors significantly complicates the process of drawing causal conclusions between exposure and outcomes. Theoretically, the MR method can effectively mitigate the impact of confounding factors and negate the influence of reverse causality. By employing a 2-sample MR approach, we explored the genetic-level causal relationship between SHBG and sarcopenia, thereby contributing positively to advancing the understanding of these 2 conditions.

## 2. Materials and methods

### 2.1. Study design

**Figure 1.**
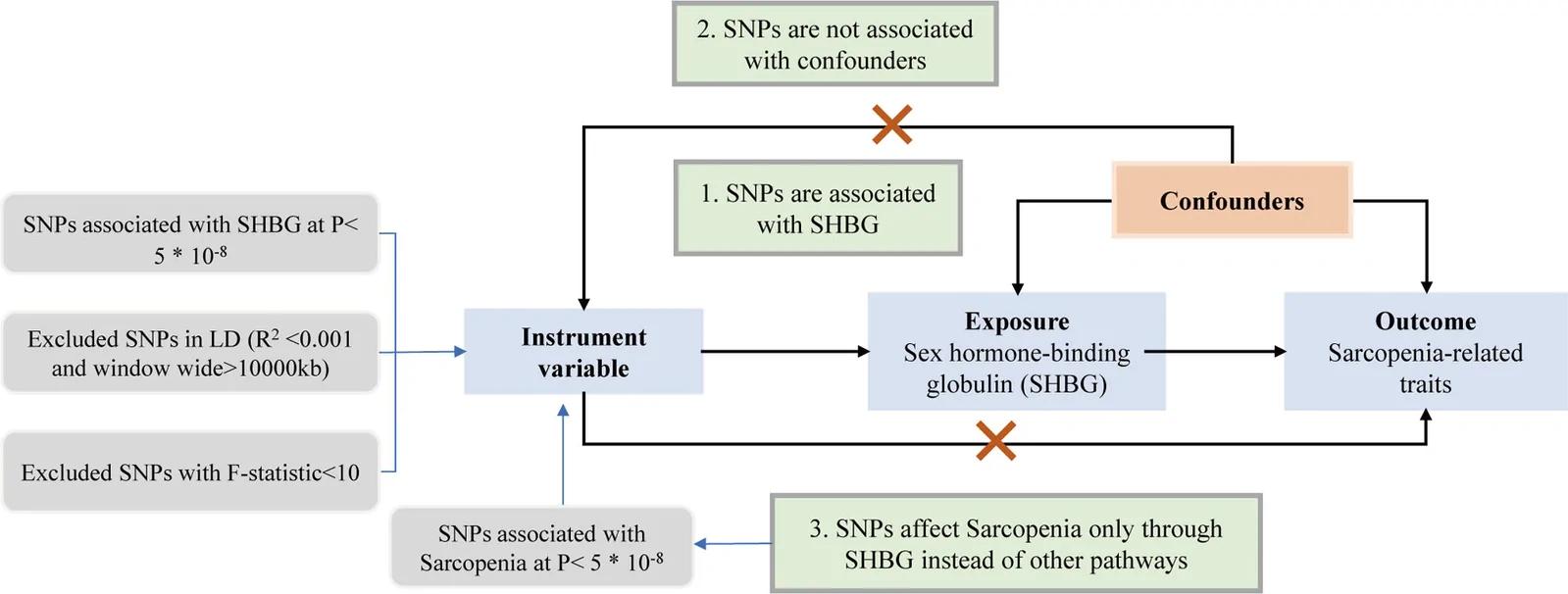
Study design overview. LD = linkage disequilibrium, SHBG = sex hormone-binding globulin, SNP = single-nucleotide polymorphism.

MR analysis, hinging upon the IV methodology that employs genetic polymorphisms as IVs, constitutes a powerful tool in discerning causal relationships in epidemiological research.^[22]^ Summary statistics on SHBG and sarcopenia from genome-wide association studies (GWAS) were obtained from public databases. This sophisticated approach is underpinned by 3 assumptions, meticulously ensuring the validity and robustness of inferred causal inferences.^[23]^ We select genetic variants that meet the following conditions for MR analysis: relevance: Select SNPs that were associated with significant genome-wide exposure in GWAS summary data; independence: SNPs are not associated with any confounders of the exposure-outcome relationship; exclusion restriction principle: the genetic variant exclusively impacts the outcome via the specific risk factor of interest. The complete process flow chart of this study is shown in Figure 1.

### 2.2. GWAS summary data source

The datasets pertaining to sex hormone-related traits utilized in our research were sourced from the IEU Open GWAS database. The summary statistics for both SHBG and body mass index (BMI)-adjusted SHBG were obtained from a comprehensive GWAS encompassing 3,70,125 individuals of European ancestry from the UK Bio-bank (UKB),^[24]^ all of whom had given their written informed consent. Further details are available in an earlier published study.^[24]^

Our study included 3 functional parameters as relevant features of sarcopenia, including usual walking pace, appendicular lean mass (ALM), and handgrip strength (left and right), as they are reliable predictors of sarcopenia.^[4]^ The GWAS data for ALM, a widely adopted proxy for muscle mass in sarcopenia research, were derived from a meta-analysis involving 4,50,243 participants from the UKB.^[25]^ ALM was assessed through bioelectrical impedance analysis.^[26]^ Reduced physical performance is a hallmark of sarcopenia, prompting the use of walking pace as a safe and dependable diagnostic tool for sarcopenia in clinical settings.^[4]^ Combined genetic predictors of usual walking pace were sourced from the UK Biobank, encompassing 4,59,915 participants. Additionally, grip strength, a prevalent measure of muscle health, reliably indicates overall muscle strength.^[4]^ In this research, absolute grip strength was used as a proxy for muscle strength. The GWAS summary-level data of total grip strength, encompassing 4,61,089 and 4,61,026 samples for left and right hands respectively, were obtained from the UKB database (https://www.ukbiobank.ac.uk/researchers/). Information for all GWAS data was detailed in Table 1.

**Table 1:** The detailed data information of the Mendelian randomization study on the association of sex hormone-binding globulin and sarcopenia.

	Consortium/study	Ancestry	Sample size	PMID	Sex (female %)	Age (yr)
Exposure						
SHBG	ebi-a-GCST90012111	European	3,70,125	32042192	51.2%	~40–69
BMI-adjusted SHBG	ebi-a-GCST90012110	European	3,70,125	32042192	51.2%	~40–69
Outcome						
ALM	GCST90000025	European	4,50,243	33097823	54.4%	48–73
Hand grip strength (left)	ukb-b-7478	European	4,61,089	–	~54%*	~40–69*
Hand grip strength (right)	ukb-b-10215	European	4,61,026	–	~54%*	~40–69*
Usual walking pace	ukb-b-4711	European	4,59,915	–	~54%*	~40–69*

ALM = appendicular lean mass, BMI = body mass index, GWAS = genome-wide association study, SHBG = sex hormone-binding globulin.

* For traits sourced directly from the MRC IEU OpenGWAS database, specific sex and age breakdowns were not available. The reported values reflect the overall characteristics of the UK Biobank cohort.

The data used in this study were obtained from publicly available databases, and the original studies had received ethical approval. As only summary-level data were utilized, no additional ethical approval was required for this analysis.

### 2.3. IVs selection

Firstly, independent SNPs associated with SHBG and BMI-adjusted SHBG were selected for analysis (*P* < 5 × 10^−8^). Secondly, SNPs with linkage disequilibrium (*R*^2^ < 0.001) and close SNPs (window size = 10,000 kb) were excluded.^[27]^ Thirdly, SNPs with a minor allele frequency below 0.01 were also excluded from the analysis. Fourthly, if there is no corresponding SNP in the obtained GWAS, the proxy SNP with a higher linkage disequilibrium relationship with the missing SNP is selected using the criterion that *R*^2^ is >0.8.^[28]^ Furthermore, we calculate the *F* statistic for the IVs by using the following formula: *F* = *R*^2^ × (N − 2)/(1 − *R*^2^), *R*^2^ denotes the variance of exposure explained by each IV. As a rule of thumb, if the average *F* statistic is >10, it can be considered that there is no weak tool bias.^[29]^ We utilized these carefully chosen SNPs as the final genetic IVs for the subsequent MR analysis.

### 2.4. Analysis of MR

In our study, we conducted the MR analysis using 4 diverse approaches: primarily through the random-effects inverse-variance weighted (IVW) method,^[30]^ with MR-Egger regression,^[31]^ weighted median (WM),^[32]^ and weighted mode^[33]^ serving as supplementary techniques. The IVW method combined the Wald ratio estimates of causal effects across different variants, assuming all IVs were valid.^[30]^ The MR-Egger method does not rely on nonzero mean pleiotropy but sacrifices some statistical power.^[31]^ However, it may lack precision and be prone to the influence of outlier genetic variants. The WM approach offers robust estimates of effective IVs, considering those with at least 50% weight.^[32]^ Meanwhile, the weighted Mode method assesses the causal effect of the subset containing the highest number of SNPs by grouping them based on the similarity of their causal effects.^[33]^ Detailed descriptions of these methods have been published elsewhere.^[34]^

### 2.5. Sensitivity analysis

The study evaluated the presence of horizontal pleiotropy utilizing both the MR-Egger intercept test^[31]^ and the Mendelian randomization-Pleiotropy Residual Sum and Outlier (MR-PRESSO) global test.^[35]^ After excluding outliers identified by the MR-PRESSO global test, a robustness analysis was conducted using the leave-one-out method to further validate the results. A significance level of *P* < .05 was established for the analysis. Additionally, the asymmetry of the funnel plot served as an indicator of potential horizontal pleiotropy.^[36]^ To quantify heterogeneity, we employed both the IVW method and MR-Egger regression, utilizing Cochran’s Q statistic. Furthermore, a leave-one-out analysis was conducted to assess the robustness and consistency of our findings. This analysis was also used to investigate whether the causal relationship between SHBG and sarcopenia could be influenced by any single SNP. All analyses were carried out using package “TwoSampleMR” in R version 4.0.5.

## 3. Results

### 3.1. IVs selection

In this study, we identified 354 and 420 independent SNPs with significant *P*-values as IV SNPs for SHBG and BMI-adjusted SHBG, respectively (Table S1, Supplemental Digital Content, https://links.lww.com/MD/Q131). During data harmonization, a small number of these SNPs could not be found in the outcome datasets and no suitable proxies were available. These SNPs were therefore excluded from the respective analyses. For the SHBG analysis, a total of 1 to 11 SNPs were removed depending on the outcome trait. For the BMI-adjusted SHBG analysis, 1 to 12 SNPs were removed. A comprehensive list of the excluded SNPs for each exposure-outcome pair is provided in Table S2, Supplemental Digital Content, https://links.lww.com/MD/Q132. The *F*-statistic values for all remaining IVs were all more than 10.

### 3.2. Mendelian randomization analysis

The Table 2 displays the MR estimates obtained from various methodologies. According to the IVW results, a causal link has been established between genetically predicted SHBG levels and an individual’s usual walking pace (odds ratio = 1.04057, 95% confidence interval: 1.01542–1.06635, *P* = .00144; Table 2). In addition, the weighted mode method showed that SHBG was associated with ALM (0.94199, 0.89134–0.99552, 0.03479; Table 2). But there were no significant relationships between SHBG and hand grip strength (Table 2). Such associations were consistent in MR-Egger, WM, and weighted model (Table 2). However, the results had not find a significant causal association between genetically predicted BMI-adjusted SHBG and sarcopenia-related traits (Table 2).

**Table 2:** MR estimates of the relationship of genetically predicted sex hormone-binding globulin and sarcopenia.

Exposure	Outcome	SNPs	Methods	OR (95% CI)	*P* value
SHBG	ALM	345	IVW	1.02151 (0.92057–1.13351)	.68852
		345	MR-Egger	1.1438 (0.95197–1.37427)	.15234
		345	Weighted median	1.03014 (0.97415–1.08934)	.29767
		345	Weighted mode	0.94199 (0.89134–0.99552)	.03479
	Hand grip strength (left)	336	IVW	1.00328 (0.97052–1.03716)	.84653
		336	MR-Egger	1.0345 (0.97479–1.09787)	.26429
		336	Weighted median	0.99294 (0.96285–1.02397)	.65196
		336	Weighted mode	0.99923 (0.96316–1.03665)	.96733
	Hand grip strength (right)	336	IVW	1.00939 (0.97523–1.04474)	.59479
		336	MR-Egger	1.0368 (0.97479–1.10276)	.25158
		336	Weighted median	0.97814 (0.94737–1.00991)	.17532
		336	Weighted mode	0.98354 (0.94232–1.02656)	.44782
	Usual walking pace	336	IVW	1.04057 (1.01542–1.06635)	.00144
		336	MR-Egger	0.96636 (0.92582–1.00867)	.11851
		336	Weighted median	1.00201 (0.97451–1.03029)	.88751
		336	Weighted mode	0.9713 (0.94019–1.00343)	.08038
BMI-adjusted SHBG	ALM	409	IVW	1.06013 (0.96436–1.1654)	.22676
		409	MR-Egger	1.10238 (0.94178–1.29037)	.2257
		409	Weighted median	0.96735 (0.91593–1.02165)	.23354
		409	Weighted mode	0.96238 (0.90985–1.01794)	.18128
	Hand grip strength (left)	398	IVW	1.00497 (0.97547–1.03537)	.74423
		398	MR-Egger	1.02103 (0.97093–1.07371)	.41802
		398	Weighted median	0.99754 (0.96638–1.02971)	.87904
		398	Weighted mode	0.98126 (0.93664–1.02801)	.42615
	Hand grip strength (right)	398	IVW	1.0069 (0.97625–1.03852)	.66273
		398	MR-Egger	1.02975 (0.9774–1.0849)	.27139
		398	Weighted median	0.97058 (0.94033–1.0018)	.06453
		398	Weighted mode	0.95193 (0.90532–1.00095)	.05519
	Usual walking pace	398	IVW	1.00079 (0.97868–1.02339)	.945
		398	MR-Egger	1.0275 (0.98958–1.06687)	.15811
		398	Weighted median	1.01012 (0.98416–1.03675)	.4485
		398	Weighted mode	0.99668 (0.95416–1.0411)	.88134

ALM = appendicular lean mass, BMI = body mass index, CI = confidence interval, IVW = inverse variance weighted, MR = Mendelian randomization, OR = odds ratio, SHBG = sex hormone-binding globulin, SNPs = single nucleotide polymorphisms.

### 3.3. Sensitivity analysis

The Cochran’s *Q* statistic, when applied in the context of the MR-IVW method, indicated the presence of heterogeneity (*P* < .05) in the MR analyses involving SHBG and SHBG adjusted for BMI, across all sarcopenia-related traits (as detailed in Table 3). Additionally, the intercept test conducted within the MR-Egger analysis revealed evidence of horizontal pleiotropy (*P* < .05) in the MR studies examining the relationship between SHBG and usual walking pace, while no horizontal pleiotropy between SHBG and other sarcopenia-related traits (*P* > .05; Table 3).

**Table 3:** Sensitivity analysis of the Mendelian randomization analysis results of sex hormone-binding globulin and sarcopenia.

Exposure	Outcome	Heterogeneity		Pleiotropy	
		*Q* statistic (IVW)	*P* value	MR-Egger Intercept	*P* value
SHBG	ALM	9777.562	0	−0.002	.144
	Hand grip strength (left)	1514.267	4.03E−149	−4.59E−04	.224
	Hand grip strength (right)	1622.852	1.06E−167	−4.02E−04	.305
	Usual walking pace	1130.778	6.40E−87	0.001	5.67E−05
BMI-adjusted SHBG	ALM	10,263.28	0	−5.89E−04	.543
	Hand grip strength (left)	1525.725	9.07E−132	−2.34E−04	.444
	Hand grip strength (right)	1638.19	4.23E−150	−3.32E−04	.296
	Usual walking pace	1178.934	1.56E−78	−3.89E−04	.089

ALM = appendicular lean mass, IVW = inverse variance weighted, MR = Mendelian randomization, SHBG = sex hormone-binding globulin.

**Figure 2.**
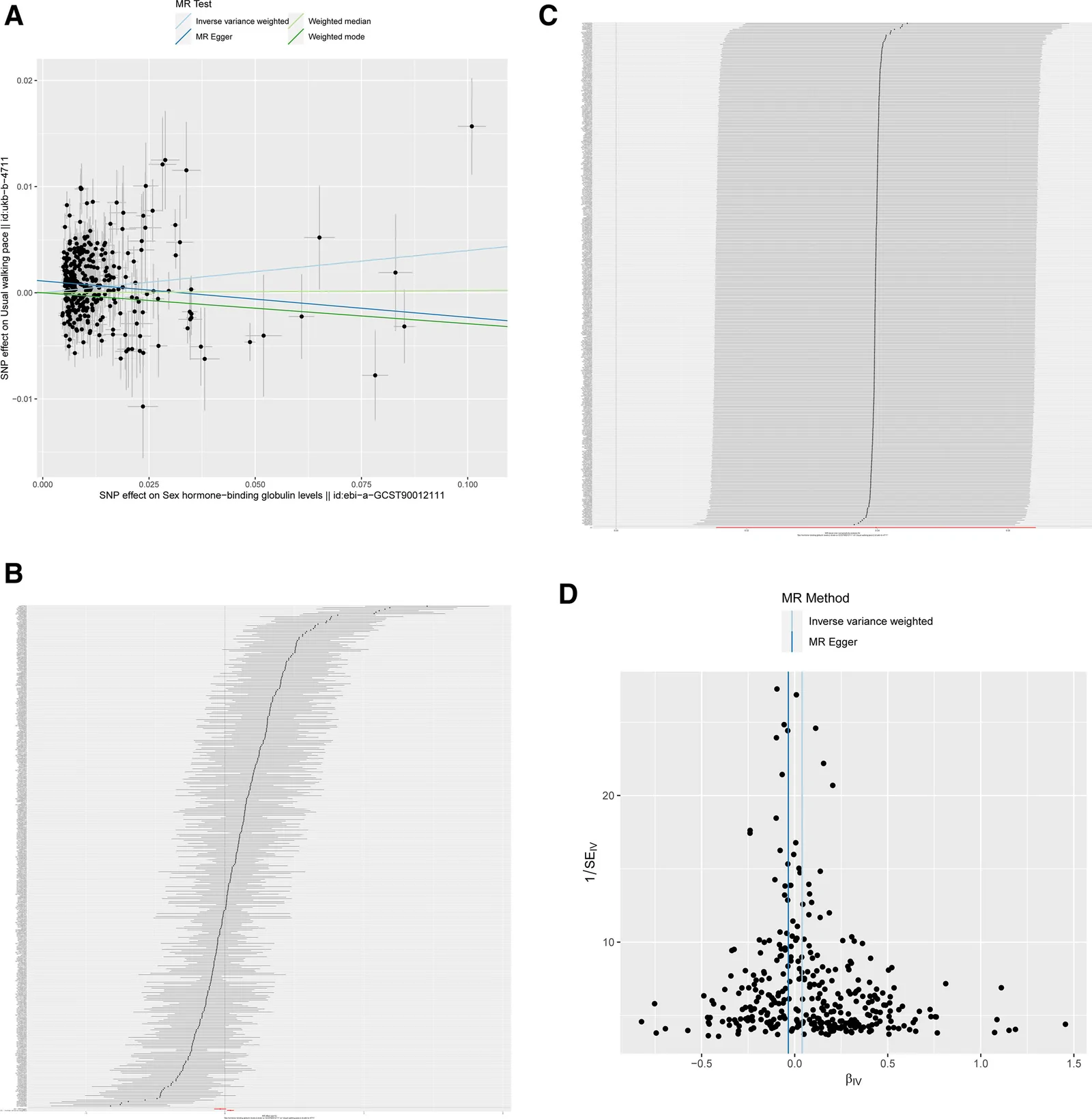
MR estimates the impact of SHBG on usual walking pace. (A) Scatter plot of the causal relationships between SHBG and usual walking pace. (B) MR leave-one-out sensitivity analyses for SHBG and usual walking pace. (C) Forest plot of the causal relationships between SHBG and usual walking pace. (D) Funnel plot of the causal relationships between SHBG and usual walking pace. MR = Mendelian randomization, SHBG = = sex hormone-binding globulin.

The global test of MR-PRESSO showed horizontal pleiotropy in MR Analyses of SHBG and BMI-adjusted SHBG with all sarcopenia-related traits (*P* < .05; Table S3, Supplemental Digital Content, https://links.lww.com/MD/Q132). The distortion test of MR-PRESSO analysis showed that the MR analyses of BMI-adjusted SHBG and ALM had outliers (Table S3, Supplemental Digital Content, https://links.lww.com/MD/Q132). The scatter plot for effect sizes of SNPs for SHBG and BMI-adjusted SHBG with sarcopenia were shown in Figures 2 and S1–S7, Supplemental Digital Content, https://links.lww.com/MD/Q133. Upon visually examining the IVW forest plot, no evidence was found to suggest that the causal estimate was disproportionately influenced by the outcome of a single variant; furthermore, the funnel plot was symmetry, indicating no pleiotropy (Figs. 2 and S1–S7, Supplemental Digital Content, https://links.lww.com/MD/Q133). The results of the leave-one-out analysis suggested that no single SNP was responsible for driving the outcomes of our MR analyses (Figs. 2 and S1–S7, Supplemental Digital Content, https://links.lww.com/MD/Q133).

## 4. Discussion

Our study marked the inaugural investigation into the connection between SHBG and sarcopenia through the application of multiple, complementary MR methods. The findings from our 2-sample MR analysis revealed an association between genetically predicted SHBG and sarcopenia-related traits. However, when adjusting for BMI, no statistically significant causal link was observed between genetically predicted SHBG and sarcopenia.

This study found a significant causal association between SHBG and sarcopenia related traits. However, there is a lack of observational studies and randomized controlled trials studies exploring the association between SHBG and sarcopenia. The main function of SHBG is to regulate the levels of free sex hormones in the body and, by binding these hormones, control their bioavailability. Changes in SHBG levels can affect the action of sex hormones, which may affect a variety of physiological processes, including muscle mass and function.^[15]^ The current research on the association between SHBG and sarcopenia is insufficient, but there are some preliminary studies pointing to a possible link. Most studies suggest that reduced SHBG levels may lead to increased levels of free testosterone, which is a key hormone for maintaining muscle mass and strength.^[10,11]^

Other studies have found that lower SHBG levels may be associated with a reduction in muscle mass and strength.^[37]^ Furthermore, SHBG levels have been linked to metabolic diseases such as polycystic ovary syndrome, and patients with these diseases may indirectly affect muscle health by affecting metabolic pathways.^[38]^ In addition, studies have found that chronic inflammation is closely related to reduced SHBG levels, and inflammation may affect muscle health through a variety of mechanisms, including the breakdown of muscle proteins.^[39]^

However, this study found that the causal connection between BMI-adjusted SHBG levels and the phenotype associated with sarcopenia became insignificant. This suggests the relationship is likely mediated or confounded by adiposity. Several factors, such as nutritional status and lifestyle, have an impact on SHBG levels. Obesity may lead to lower SHBG levels, and proper diet and exercise may help maintain healthy SHBG levels, which may have a positive impact on muscle health.

Previous researches have noted that being overweight or obese as measured by BMI is inversely linked with the risk of sarcopenia.^[37,40,41]^ However, Liu et al found that this negative association could be influenced by muscle mass, and that after adjusting for muscle mass, a higher BMI was associated with an increased risk of sarcopenia.^[41]^ This is partly related to the positive association between visceral fat area and sarcopenia risk, suggesting that excess fat alone is not a protective factor for sarcopenia.^[42]^ In contrast, sarcopenic obesity, which affects 11% of older adults globally, is linked with a variety of adverse outcomes.^[43]^ The correlation mechanism and possible influencing factors between SHBG and sarcopenia need to be further explored in the follow-up researches.

A notable strength of this research lies in its successful application of the MR method, which significantly minimizes the influence of reverse causality and confounding variables. More importantly, this study explores the causal association between SHBG and sarcopenia at the genetic level, covering the broadest population, and is more practical and convincing than traditional observational studies.

However, our study also has several limitations. First, considering muscle mass and strength, the genetic structure of sarcopenia remains unclear,^[44]^ the SNPs selected in this study have the potential to be inconsistent in their effects on traits, making it difficult to select appropriate IVs to act as true proxies for exposure. Therefore, research into the function of SNPs may be needed to help explain the genetic characteristics of SHBG and sarcopenia, and to facilitate the selection of more precise IVs. Secondly, the genetic evaluation of the effect of SHBG and sarcopenia in this study often ignores the influence degree of environmental factors, which may only partially explain the effect of SHBG and sarcopenia.

## 5. Conclusion

In brief, the existing 2 sample MR study indicates a link between genetically determined SHBG and sarcopenia. These findings offer fresh perspectives on the genetic mechanisms underlying SHBG and sarcopenia, warranting additional research using larger GWAS datasets to corroborate these observations. In addition, the potential gene-environment interactions in sarcopenia are worth exploring.

## Author contributions

**Conceptualization:** Moyan Liu, Lichao Zhao, Xiaoping Sun, Jie Xu, Zhaoyan Zhang.

**Data curation:** Moyan Liu, Lichao Zhao, Yan Gao, Juan Hui.

**Writing – original draft:** Moyan Liu, Lichao Zhao, Yan Gao, Xiaoping Sun, Jie Xu, Juan Hui, Peng Xu, Qingfeng Yu, Zhaoyan Zhang.

**Writing – review & editing:** Moyan Liu, Lichao Zhao, Yan Gao, Xiaoping Sun, Jie Xu, Juan Hui, Peng Xu, Qingfeng Yu, Zhaoyan Zhang.

## Supplementary Material
