# Supplementary material for: The causal effects of genetically predicted sex hormone-binding globulin and sarcopenia: A 2-sample Mendelian randomization study
Source: Medicine (Baltimore). 2025 Sep 26;104(39):e44654. doi: 10.1097/MD.0000000000044654 (PMC13592953; doi:10.1097/MD.0000000000044654)
Supplement: Supplementary file 1 [file medi-104-e44654-s002.pdf]

Table S2 Proxy SNP situation.

**Exposure**	**Outcomes**	**Unfound SNPs**	**Target SNP**	**Proxy SNP**	**Unfound Proxies**
SHBG	Hand grip strength (left)	11("rs28549287" "rs113430755" "rs13031661" "rs3958042" "rs6940266" "rs10794307" "rs8049669" "rs10401031" "rs8071851" "rs190408119" "rs202200760")			11
	Hand grip strength (right)	11 ("rs28549287" "rs113430755" "rs13031661" "rs3958042" "rs6940266" "rs10794307" "rs8049669" "rs10401031" "rs8071851" "rs190408119" "rs202200760")			11
	Usual walking pace	11 ("rs28549287" "rs113430755" "rs13031661" "rs3958042" "rs6940266" "rs10794307" "rs8049669" "rs10401031" "rs8071851" "rs190408119" "rs202200760")			11
	ALM	1("rs11110390")			1
BMI-adjusted SHBG	Hand grip strength (left)	12 ("rs28549287" "rs185241389" "rs58304838" "rs58936320" "rs11130982" "rs376263969" "rs9272718" "rs78816009" "rs112924565" "rs193159515" "rs202200760" "rs7289655")			12
	Hand grip strength (right)	12 ("rs28549287" "rs185241389" "rs58304838" "rs58936320" "rs11130982" "rs376263969" "rs9272718" "rs78816009" "rs112924565" "rs193159515" "rs202200760" "rs7289655")			12
	Usual walking pace	12 ("rs28549287" "rs185241389" "rs58304838" "rs58936320" "rs11130982" "rs376263969" "rs9272718"			12

		"rs78816009" "rs112924565" "rs193159515" "rs202200760" "rs7289655")			
	ALM	1 ("rs11110390")			1

ALM, Appendicular lean mass; SHBG, Sex hormone-binding globulin levels

Table S3. MR-PRESSO results of the causal relationship between genetically predicted sex hormone-binding globulin and sarcopenia.

Exposure	Outcome	Raw			Outlier corrected			Global *p*	Outliers	Distortion *p*
		OR	*95%CI*	*P*	OR	*95%CI*	*P*			
SHBG	ALM	1.02	(1.131-0.921)	0.7	1.024	(1.074-0.977)	0.323	<0.001	130	0.838
	Hand grip strength (left)	1.001	(1.035-0.969)	0.932	0.994	(1.021-0.969)	0.672	<0.0003	27	0.1796667
	Hand grip strength (right)	1.007	(1.042- 0.973)	0.697	0.984	(1.011- 0.959)	0.24	<5e-04	37	0.2665
	Usual walking pace	1.038	(1.064-1.013)	0.003	1.038	(1.060-1.017)	5.36E-04	<0.001	22	0.988
BMI-adjusted SHBG	ALM	1.055	(1.158-0.9617)	0.257	1.025	(1.072-0.980)	0.288	<0.001	145	0.003
	Hand grip strength (left)	0.9997	(1.0296-0.971)	0.986	0.9876	(1.012-0.964)	0.3122	<0.001	31	0.698
	Hand grip strength (right)	1.0009	(1.032-0.9708)	0.9495	0.9703	(0.995-0.9468)	0.0172	<0.001	32	0.424

	Usual walking pace	0.9991	(1.0214- 0.9774)	0.9412	0.9957	(1.015- 0.9766)	0.6659	<0.001	24	0.837
